# Comparison of the labor curves with and without combined spinal-epidural analgesia in nulliparous women- a retrospective study

**DOI:** 10.1186/s12884-020-03161-x

**Published:** 2020-08-15

**Authors:** Hitomi Ando, Shintaro Makino, Jun Takeda, Yojiro Maruyama, Shuko Nojiri, Hiroyuki Sumikura, Atsuo Itakura

**Affiliations:** 1 Department of Obstetrics and Gynecology, Juntendo University Faculty of Medicine, 2-1-1 Hongo, Bunkyo-ku, Tokyo, 113-8421 Japan; 2 Medical Technology Innovation Center Clinical Research and Trial Center, Juntendo University Faculty of Medicine, Tokyo, Japan; 3 Department of Anesthesia and Pain Medicine, Juntendo University Faculty of Medicine, Tokyo, Japan

**Keywords:** Combined-spinal epidural analgesia, Labor curve, Labor management, Neuraxial labor analgesia, Primiparous, Vaginal delivery

## Abstract

**Background:**

Neuraxial labor analgesia is known to increase the rate of instrumental delivery and prolong the second stage of labor; however, there is no standard method to evaluate the progress of labor under analgesia. Friedman curve is considered the gold standard for evaluating the progress of labor. However, it included not only neuraxial labor analgesia but also labor without analgesia. Thus we compared the labor curves of primiparous women undergoing labor with and without neuraxial labor analgesia, to understand the progress of labor in both groups and to arrive at a standard curve to monitor the progress of labor under neuraxial analgesia.

**Methods:**

Primiparous women with cephalic singleton pregnancies who delivered at term from 2016 to 2017 were included. Two hundred patients who opted for combined spinal-epidural (CSE) labor analgesia were included in the CSE group and 200 patients who did not undergo CSE were included in the non-CSE group. In all, 400 cases were examined retrospectively. The evaluation parameters were cervical dilation and fetal station, and we calculated the average value per hour to plot the labor curves.

**Results:**

The labor curve of the non-CSE group was significantly different from the Friedman curve. In the CSE group, the time from 4 cm dilation of the cervix to full dilation was 15 h; in addition, the speed of cervical dilation was different from that in the non-CSE group. The progress of labor in the CSE group was faster than that in the non-CSE group during the latent phase; however, the progress in the CSE group was slower than that in the non-CSE group during the active phase.

**Conclusions:**

Neuraxial labor analgesia results in early cervical dilation and descent of the fetal head; thus, appropriate advance planning to manage the delivery may be essential.

## Background

Vaginal delivery without neuraxial labor analgesia is the most common method for childbirth; however, the number of pregnant women opting for neuraxial labor analgesia is increasing in Japan, [1] and our facility is the leading institute providing neuraxial labor analgesia. Neuraxial labor analgesia has several challenges, such as a prolonged second stage of labor, and an increased rate of instrumental delivery [2, 3]. Hence, augmentation is required during the second stage of labor, and the timing for oxytocin augmentation is difficult to decide because there is no standard curve to assess the progress of labor under neuraxial analgesia.

In 1954, Friedman examined 500 primiparous women every hour, to create a curve showing the average time it took for the cervix to dilate each by centimeter [4]. Usually, the Friedman curve is considered the gold standard for evaluating the progress of labor. However, it has been reported that the Friedman curve differs from progress of labor in recent times [5, 6].

Another labor curve was created in 2002 after studying 1329 primiparous women, it is called Zhang curve, and it is significantly different from the Friedman curve [7]. According to this curve, the cervical dilation was significantly slower in the active phase. Moreover, the Zhang curve was not observed during the deceleration phase. According to the Zhang curve, for the cervical dilation to increase from 4 cm to 10 cm, the duration was extended to approximately 5.5 h in contrast to 2.5 h reported in the Friedman curve.

In Japan, the progress of spontaneous labor in primiparous women has also been reported by Suzuki et al. [8]. The labor curve reported by them for the cervical dilation to increase from 4 cm to 10 cm extended to approximately 5 h, thus the progress of labor in primiparous in Japanese women was considered to be slow paced.

Interestingly, in Friedman’s study, 8% of the cases underwent neuraxial labor analgesia. Similarly, the study on which the Zhang curve was based also comprised 48% cases that underwent neuraxial labor analgesia.

However, there is no standard tool to evaluate the progress of labor under neuraxial analgesia. Therefore, we conducted this study to evaluate the labor curve under neuraxial labor analgesia.

We aimed to understand the progress of labor in primiparous women who opted for CSE analgesia.

## Methods

### Study population

This was a retrospective study and the data was collected from the medical records from January 2016 to December 2017 in the Juntendo university hospital in Tokyo, Japan. The study population comprised two groups-one that underwent combined spinal-epidural analgesia (CSE group) and the second group that underwent without neuraxial labor analgesia (non-CSE group). The inclusion criteria of both groups were (1) primiparous women with cephalic singleton pregnancies, (2) full term births (between 37 weeks, 0 days to 41 weeks, 6 days), and (3) vaginal delivery. In the CSE group, neuraxial labor analgesia was initiated in the first stage of labor for women who opted for CSE analgesia. Multiple pregnancy, multiparous women, cesarean delivery (scheduled and emergency) were excluded from both groups. In the CSE group, patients receiving only epidural analgesia, patients receiving CSE twice because of less effect, and patients opting for CSE in the second stage of labor were also excluded. Two hundred cases who underwent CSE were collected in the CSE group and 200 cases who did not undergo CSE collected as the non-CSE group. The subjects in the non-CSE group were extracted by matching the gestational age, BMI, and neonatal birth weight to those in the CSE group. In all, 400 cases were examined retrospectively. This study was approved by Ethics Committee of Juntendo University Hospital (No.19–102).

### Labor management

The pelvic examination for assessing the cervical dilation and fetal station was performed by obstetricians or midwifes. The frequency of examination depended on the progress of labor. Oxytocin augmentation was used when the labor was clinically thought to be arrested over 1 h. CSE analgesia was administered by obstetric anesthesiologists. CSE analgesia was administered on the patient’s request after the onset of labor.

The CSE analgesia comprised injection of bupivacaine 0.5% 0.5 ml (2.5 mg) and fentanyl 0.2 ml (10 μg), mixed with physiological saline 1.3 ml into the subarachnoid space, and epidural analgesia was administered by a patient-controlled epidural anesthesia pump. This consisted of 1 mg/ml levobupivacaine 0.08% and fentanyl 2 μg / ml (total 5 ml) with a lockout time of 15 min by bolus administration.

### Data analysis

The evaluation parameters were cervical dilation and fetal head station, and the mean value per hour was calculated to construct the labor curves.

The active phase and latent phase of labor were defined as per WHO recommendations [9]. The latent phase is the period characterized by painful uterine contractions and variable changes in the cervix, including some degree of effacement and slower progression of dilation up to 5 cm. The active phase is a period characterized by regular painful uterine contractions, a substantial degree of cervical effacement, and more rapid cervical dilation from 5 cm until full dilation.

Demographic variables were analyzed using descriptive statistics. The outcomes were evaluated using the unpaired Student’s t-test, chi-square test, and Fisher’s exact test as appropriate. A value of *p* < 0.05 was considered statistically significant. SAS 9.4 software (SAS Institute, Cary, NC) was used for all statistical analyses.

The regression line between CSE and non-CSE group for cervical dilation was compared by F test for time and cervical dilation as an interaction term in < 5 cm (the latent phase)and > = 5 cm (the active phase). We also calculated approximations for the cervical dilation curves using Excel.

## Results

The comparison of patients’ baseline data is shown in Table 1. There were no differences in the patients’ baseline data between the 2 groups, other than the mean age. The patients in the CSE group patients were significantly older than those in the non-CSE group.

**Table 1:** Comparison of characteristics between the non-CSE group and the CSE group

	Non-CSE (***n*** = 200)	CSE (***n*** = 200)	
	Mean (SD)	Mean (SD)	***p*** value
Age (years)	32 (4.6)	34 (4.9)	< 0.05
Gestational age (weeks)	39.6 (1.2)	39.7 (1.1)	0.96
BMI (kg/m^2^)	24.4 (3.1)	24.8 (3.1)	0.27
Birth weight (g)	3034 (386.8)	3065 (450.0)	0.46

*CSE* Combined spinal-epidural analgesia

*SD* Standard deviation

*P* value < 0.05 is considered significant

The comparison of delivery results is shown in Table 2. Significantly more number of patients in the CSE group required augmentation of labor and instrumental delivery than that in the non-CSE group. Moreover, there were less cases of induction and normal delivery in the CSE group than in the non-CSE group.

**Table 2:** Comparison of delivery results between the non-CSE group and the CSE group

	Non-CSE (***n*** = 200)	CSE (***n*** = 200)	
	**%(n)**	**%(n)**	***p*** **value**
Spontaneous	54.5 (109)	24.0 (48)	< 0.05
Induction	21.0 (42)	9.5 (19)	< 0.05
Augmentation	9.0 (18)	41.0 (82)	< 0.05
Normal delivery	79.0 (158)	51.5 (103)	< 0.05
Instrumental delivery	21.0 (42)	48.5 (97)	< 0.05

Spontaneous: vaginal delivery without induction or augmentation or instrumental

Normal delivery: vaginal delivery without instrumental

In the CSE group, the average amount of PCA used was 49.9 ml in the first stage of labor and 25.85 ml in the second stage of labor, which was high in the first stage.

**Fig. 1:**
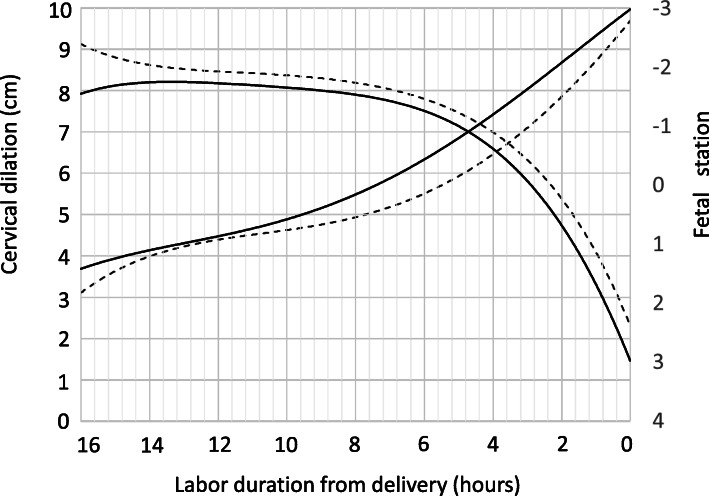
Comparison of the labor curves between the CSE group and the non-CSE group. Solid line = CSE group; Dotted line = non-CSE group.

Figure 1 demonstrates the comparison of labor curves of both groups. The solid line shows the degree of cervical dilation and the fetal station in the CSE group, and the dotted line shows the data for non-CSE group. The horizontal axis was plotted as 0 h at the time of delivery, and was traced back to 16 h at an interval of every 2 h.

The labor curve of non-CSE group was significantly different from the Friedman curve; the acceleration and deceleration phases were not observed, and a slower progression in cervical dilation from 4 cm to full dilation was noted.

In the CSE group, the time taken from cervical dilation of 4 cm to full di1lation was 15 h. In addition, the speed of cervical dilation in this group was different from that of non-CSE group.

The quadratic approximation of cervical dilation curve was as follows.

CSE group: *y* = 0.0214 × ^2^–0.7223 *x* + 9.9632.

Non-CSE group: *y* = 0.0235 *×* ^2^–0.7212 *x* + 9.2731.

The comparison of the regression lines between CSE and non-CSE group for cervical dilation up to 5 cm was not parallel (*p* < 0.005). In the same way, the comparison of the regression lines between CSE and non-CSE group for cervical dilation from 5 cm to 10 cm was not parallel (*p* = 0.008). Both of the regression lines were statistically significant interaction.

Thus the progress of labor in the CSE group was faster than that in the non-CSE group during the latent phase; however, the progress in the CSE group was slower than that in the non-CSE group during the active phase.

## Discussion

We studied the pattern of progress of labor under neuraxial analgesia. According to the labor curve, the speed of cervical dilation of the CSE group is different from that of the non-CSE group.

Neuraxial labor analgesia accelerates the initiation of cervical dilation, and it is thought that the descent of the fetal head occurs rapidly. CSE analgesia is known to induce rapid cervical dilation [10]. However the influence of uterine contraction on cervical dilation is not significant, and the progress of labor is slow [11]. This might be due to the relaxation of tension in the pelvic floor muscles once the neuraxial analgesia is administered. Moreover, the speed of cervical dilation might be affected by the function of the autonomic nervous system associated with the uterus [12].

The labor curve with neuraxial analgesia has previously been reported by Frigo et al. in 2011, but the shape of the curve was quite different from that of the curve seen in our study [13]. The difference could be because Frigo et al. included the time from examination of the patient before the administration of neuraxial labor analgesia. Our labor curve was plotted from the point of time after the administration of neuraxial labor analgesia.

It is thought that the rate of rotational abnormalities and arrest of labor increase with neuraxial labor analgesia [14, 15]. Furthermore, a high fetal head station (+ 2 ~ + 3) and malrotation were reported to be risk factors for severe lacerations during instrumental delivery [16].

By understanding the labor curve under neuraxial labor analgesia, it is possible to plan the management of cases in advance including augmentation and instrumental delivery at an appropriate time.

This study has some limitations. The additional top ups through the epidural route was not considered; however, we presume that this factor had little effect on the results since the amount administered was small. Although our sample size was small compared to that of Friedman’s study, a significant difference in the curves was recognized. Number of inductions were less in the CSE group and more in the non-CSE group, it may affect the labor curves.

## Conclusion

Neuraxial labor analgesia induced early cervical dilation and descent of the fetal head, and thus, an appropriate management of the progress of labor needs to be planned in advance.

## Data Availability

The datasets used and/or analyzed during the current study are available from the corresponding author on reasonable request.

## Abbreviations

CSE: Combined spinal-epidural analgesia
GA: Gestational age
OR: Odds ratio
SD: Standard deviation
WHO: World health organization
